# Development of Emerin mRNA Lipid Nanoparticles to Rescue Myogenic Differentiation

**DOI:** 10.3390/ijms26167774

**Published:** 2025-08-12

**Authors:** Nicholas Marano, Liza Elif Guner, Rachel S. Riley, James M. Holaska

**Affiliations:** 1 Department of Biomedical Sciences, Cooper Medical School of Rowan University, Camden, NJ 08103, USA; maranon@rowan.edu; 2 Rowan-Virtua School of Translational Biomedical Engineering and Sciences, Rowan University, Stratford, NJ 08084, USA; rileyr@rowan.edu; 3 Department of Biomedical Engineering, Henry M. Rowan College of Engineering, Rowan University, Glassboro, NJ 08028, USA; gunerl47@students.rowan.edu

**Keywords:** Emery–Dreifuss muscular dystrophy, EDMD, emerin, lipid nanoparticle, myogenic differentiation

## Abstract

Emery–Dreifuss muscular dystrophy 1 (EDMD1) arises from mutations in *EMD*. Most EDMD1 patients lack detectable emerin expression. They experience symptoms such as skeletal muscle wasting, joint contractures, and cardiac conduction defects. Currently, physicians rely on treating patient symptoms without addressing the underlying cause—lack of functional emerin protein. Thus, there is a need for therapeutic approaches that restore emerin protein expression to improve patient outcomes. One way would be to deliver emerin mRNA or protein directly to affected tissues to restore tissue homeostasis. Here, we evaluated the utility of lipid nanoparticles (LNPs) to deliver emerin mRNA to diseased cells. LNPs have been studied for decades and have recently been used clinically for vaccination and treatment of a myriad of diseases. Here, we show that the treatment of emerin-null myogenic progenitors with LNPs encapsulating emerin mRNA causes robust emerin protein expression that persists for at least 4 days. The treatment of differentiating emerin-null myogenic progenitors with 2.5 pg/cell emerin LNPs significantly improved their differentiation. The toxicity profiling of emerin mRNA LNP (EMD-LNP) dosing shows little toxicity at the effective dose. These data support the potential use of EMD-LNPs as a viable treatment option and establishes its utility for studying EDMD pathology.

## 1. Introduction

Emery–Dreifuss muscular dystrophy 1 (EDMD) is an X-linked laminopathy caused by mutations in the gene coding for the inner nuclear membrane (INM) protein emerin [1]. Although emerin is ubiquitously expressed, EDMD1 affects specific tissues, including skeletal muscle and cardiac tissue. Patients experience skeletal muscle wasting, joint contractures, and cardiac defects [2,3]. It is common for EDMD1 patients to harbor nonsense mutations in *EMD*, resulting in no detectable emerin expression.

The skeletal muscle pathology of EDMD1 arises, at least in part, from an impaired myogenic differentiation program. Emerin function is critical for myogenic differentiation [4]. Myogenic progenitors from an emerin-knockout mouse exhibited impaired differentiation with delayed cell cycle withdrawal, belated expression of myogenic markers, and impaired myotube fusion [5,6]. Myoblasts from emerin-knockout mice [7] and mouse myoblasts expressing emerin–RNAi [8] also showed defective myogenic differentiation. RNA sequencing (RNA-seq) of differentiating emerin-null H2K myogenic progenitors showed that the temporal expression of the cell cycle and myogenic lineage genes were perturbed [6]. This is likely due to loss of key emerin interactions with various binding partners, including histone modifying machinery such as HDAC3 [9], G9a and EZH2 [10], transcription regulatory proteins [11,12,13,14], and proteins that maintain nuclear architecture [15,16,17].

Clinical interventions for EDMD1 patients rely on treating symptoms. For example, joint contractures can be treated with physical therapy or surgical intervention to improve joint mobility and reduce pain [18]. Cardiac tissue is also affected in EDMD1, and treatments include angiotensin-converting enzyme (ACE) inhibitors or angiotensin receptor blockers (ARBs) to assist with arrythmias [19]. As the disease progresses, patients often require a pacemaker to maintain proper cardiac function [20,21].

Over the last decade, there have been major advances in gene therapy treatments to treat Duchenne muscular dystrophy (DMD). DMD is caused by mutations in the *DMD* gene, which codes for dystrophin. Mutations in *DMD* are most often deletion or duplication mutations that disrupt the reading frame alignment, leading to a loss of the dystrophin protein. Thus, an exon-skipping treatment, Eteplirsen, has been developed for patients with a specific mutation in exon 51. Eteplirsen is a small antisense oligonucleotide that targets the splicing machinery to exon 51 in order to remove it [22]. Other treatments have been developed that target different exons within *DMD*, such as Golodirsen [23] and Viltolarsen [24] for exon 53 and Casimersen [25] for exon 45. Recently, Elevidys, a new treatment for DMD, introduces a smaller version of dystrophin (micro-dystrophin) into the muscles of DMD patients. Importantly, Elevidys treatment was found to ameliorate the symptoms and restore skeletal muscle function. This approach utilized the Adeno-associated virus-based vector to deliver the micro-dystrophin gene to the skeletal muscle [26,27,28]. AAVs are excellent for delivering genes to cells, but they often lack tissue or cell specificity. Furthermore, AAVs tend to have high liver tropism, and past studies have resulted in high liver toxicity upon AAV treatment [29]. Elevidys used a new AAV derivative that has low liver tropism and high skeletal muscle tropism to address this and showed less liver toxicity [30,31].

Little has been performed using lipid nanoparticle-based gene therapies in DMD or other muscle disorders. Furthermore, gene therapy treatment options for EDMD1 are lacking, and there are no reports of these being developed to treat EDMD1. Thus, we sought to develop a new gene delivery method for emerin in EDMD1 skeletal muscle cells. We utilized lipid nanoparticle (LNP) technology to deliver emerin mRNA to cells [32]. LNPs have been studied extensively for nucleic acid delivery both in vitro and in vivo in cancer [33,34], viral indications [35,36], genetic diseases [37], pregnancy [38,39], and more, with several platforms having gained FDA approval [35,36,37]. To our knowledge, LNPs have not been developed or tested as a therapeutic strategy for EDMD1. Therefore, emerin mRNA LNPs represent a new treatment option. These delivery vehicles offer protection for the nucleic acid cargo during intracellular uptake and cytosolic nucleic acid delivery, overcoming many nucleic acid delivery challenges [40]. Moreover, these platforms are safe and are well tolerated in clinical studies. Therefore, LNP-mediated mRNA delivery has emerged as a promising avenue for the expression of transient proteins. Here, we encapsulated human emerin mRNA within LNPs (EMD-LNPs) and show that this platform delivers robust emerin expression in emerin-null myogenic progenitors while showing minimal toxicity. Importantly, EMD-LNPs rescue emerin-null myogenic differentiation to wildtype levels.

## 2. Results

### 2.1. LNP Synthesis and Characterization

In this study, LNPs comprised 35% C12-200 ionizable lipid, 10% DOPE, 1.5% DMPE-PEG, and 53.5% cholesterol based on our prior studies that utilized this formulation for mRNA delivery [38,39]. Here, we used the ionizable lipid C12-200 because it has been extensively evaluated for nucleic acid delivery to a variety of tissues and cell types [38,41,42,43]. The ionizable lipid component is pH-responsive and becomes protonated in acidic environments. This interaction allows for endosomal escape once taken up by cells and is the main driver of RNA encapsulation within LNPs [44]. Either luciferase or emerin mRNA was encapsulated within LNPs for experimental analysis. On average, LNPs with luciferase mRNA were 115.15 nm in diameter and had 36.58 (μg/mL) mRNA encapsulation (Figure S1A,B). Similarly, LNPs with emerin mRNA were 121.36 nm in diameter and had 33.39 (μg/mL) mRNA encapsulation (Figure S1A,B). All LNPs were highly monodisperse, with polydispersity indices below 0.2 (Figure S1C).

### 2.2. Lipid Nanoparticles Efficiently Deliver Emerin mRNA to H2K Myogenic Progenitors

**Figure 1:**
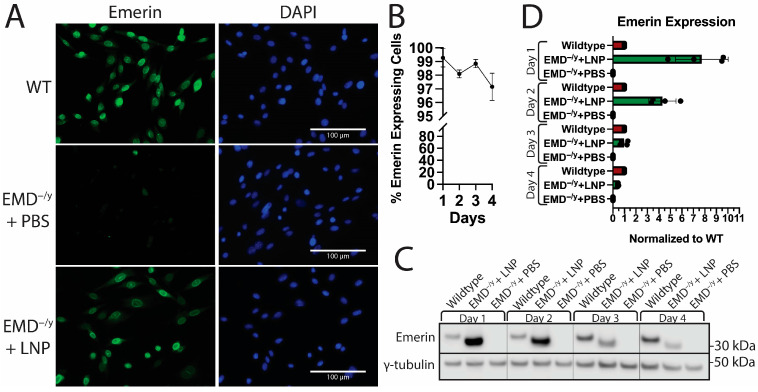
EMD-LNPs efficiently deliver cargo in vitro. (**A**) Emerin-null myogenic progenitors dosed with 2.5 pg/cell EMD-LNPs (EMD^−/y^ + LNP) or PBS (EMD^−/y^ + PBS) for 22 h. Immunofluorescence microscopy was performed using emerin antibodies (green). DAPI (blue), DNA. (**B**) Greater than 97% of EMD-LNP-treated emerin-null progenitors retain emerin protein expression after 4 days. (**C**) Western blotting was performed on wildtype (WT) or emerin-null proliferative myogenic progenitors treated with EMD-LNPs (EMD^−/y^ + LNP) or PBS (EMD^−/y^ + PBS) over four days. (**D**) Quantitation of emerin protein expression normalized to endogenous emerin in wildtype progenitors. Scale bar represents 100 µm. Error bars represent S.D. (*n* = 4).

**Figure 2:**
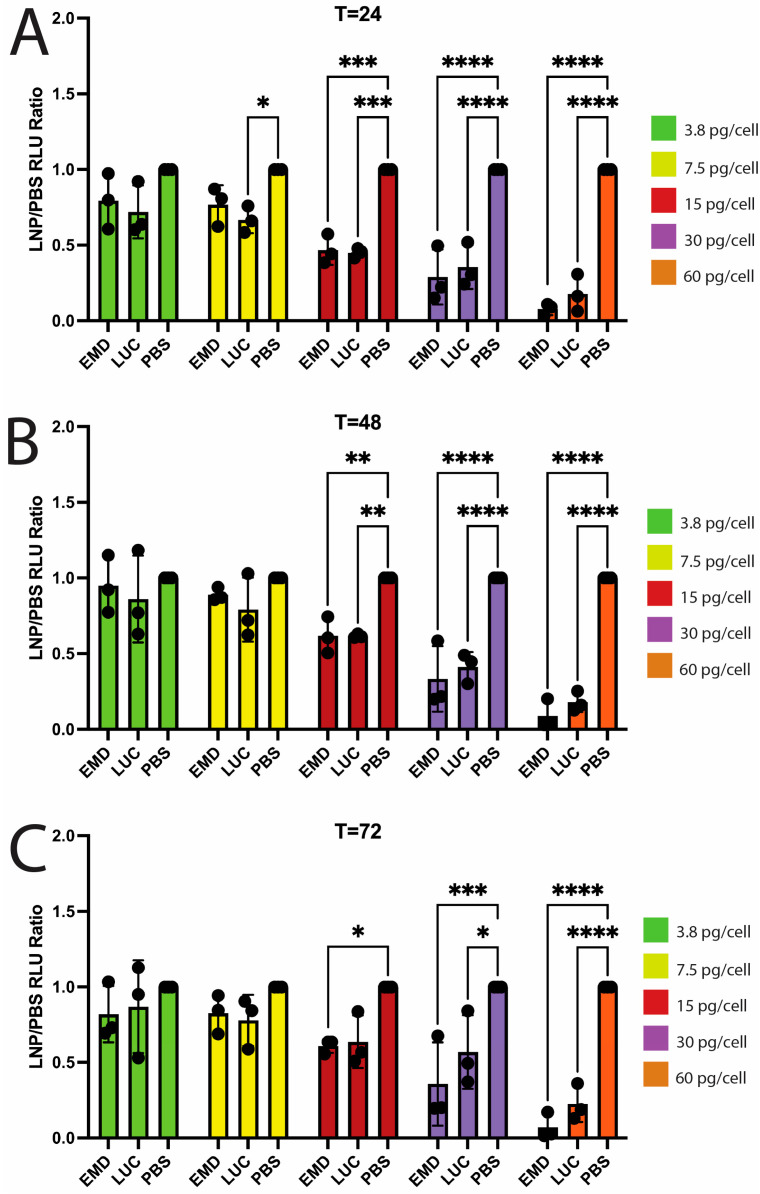
EMD-LNPs become cytotoxic at ≥15 pg/cell. PrestoBlue cell viability assays were used to measure cellular metabolism in proliferating myogenic progenitors treated with increasing concentrations of PBS (PBS), EMD-LNP (EMD), or luciferase mRNA LNP (LUC). Measurements were recorded every 24 h for 72 h, and relative light units (RLUs) were normalized to PBS: (**A**) 24 h after LNP incubation; (**B**) 48 h after LNP incubation; (**C**) 72 h after LNP incubation. Error bars represent S.D. (*n* = 3); * *p* ≤ 0.05; ** *p* ≤ 0.01; *** *p* ≤ 0.001; **** *p* ≤ 0.0001.

After incubating EMD-LNPs (2.5 pg/cell) with emerin-null myogenic progenitors for 24 h, >98% of nuclei expressed emerin (Figure 1A). In total, >95% of nuclei retained emerin expression after 96 h (Figure 1B). Emerin protein expression was measured by Western blotting, which showed increased human emerin expression following LNP treatment. The dosage was chosen based upon previous studies using a similar LNP formulation that demonstrated that 2.5 pg/cell had high protein expression and low toxicity [38,39], which we confirmed here (See Figure 2). It is important to note that the human emerin produced by the EMD-LNP mRNA migrated at a lower molecular weight than wildtype mouse emerin (Figure 1C). This is because human emerin is smaller than the mouse isoform. At 24 h after EMD-LNP treatment of emerin-null progenitors, emerin levels were 7.72-fold (±1.98) greater than endogenous emerin in wildtype myogenic progenitors (Figure 1C,D). Western blotting every day for 4 days (Figure 1C,D) showed emerin protein expression peaked at 24 h and decreased over time. At 48, 72, and 96 h after EMD-LNP dosing, emerin expression in emerin-null myogenic progenitors was 4.31 (±1.02)-fold, 0.98 (±0.29)-fold, and 0.44 (±0.03)-fold that of wildtype emerin levels, respectively (Figure 1C,D).

### 2.3. LNPs Yield No Toxicity at Effective Treatment Doses

We next determined the cytotoxic profile of LNPs containing emerin mRNA or luciferase mRNA (LUC-LNP). We utilized the PrestoBlue cell viability reagent, which measures cellular metabolism by the conversion of a non-fluorescent substrate (resazurin) into a fluorescent product (resorufin). Cells were dosed with increasing concentrations of emerin or luciferase mRNA LNPs (0–60 pg/cell) or PBS (equivalent volume). Cells were incubated with LNPs or the control for 22 h, and PrestoBlue cell viability assays were performed over 3 days (Figure 2 and Figure S2). Dosing of EMD-LNPs and LUC-LNPs <15 pg/cell were non-toxic (Figure 2 and Figure S2), which is well above the effective doses used for these studies (2.5 pg/cell). However, EMD-LNP and LUC-LNP dosing at ≥15 pg/cell had a significantly reduced signal 24 h after treatment compared to the PBS control (Figure 2A). EMD-LNP doses at 15, 30, and 60 pg/cell reduced the signal by 53.3% ± 9.7%, 71.0% ± 18.2%, and 92.3% ± 4.0%, respectively, when compared to PBS controls. While viability was significantly reduced at these doses, only at 60 pg mRNA/cell were cells non-viable, as observed by PrestoBlue (Figure 2A) and brightfield microscopy (Figure S3). To monitor LNP cytotoxicity over time, these cells were washed with PBS and replaced with proliferative media overnight. This process was repeated every 24 h for 3 days (Figure 2 and Figure S2). Significant decreases in fluorescence were also observed for both emerin and luciferase mRNA LNPs at concentrations of ≥15 pg/cell at 48 h (Figure 2B) and 72 h (Figure 2C), indicative of cytotoxicity. At 48 h after EMD-LNP dosing, cells dosed with 15, 30, and 60 pg/cell showed 38.2% ± 12.0%, 66.6% ± 21.7%, and 91.2% ± 9.8% reductions in the signal when compared to PBS controls, respectively (Figure 2B). Similarly at 72 h, 15, 30, and 60 pg/cell EMD-LNP doses led to 39.1% ± 4.7%, 64.2% ± 27.3%, and 92.9% ± 8.7% reductions in the signal compared to PBS controls, respectively (Figure 2C).

Emerin-null myogenic progenitors dosed with 2.5 pg/cell of EMD-LNPs did not exhibit cytotoxicity as expected (Figure S4A–D). A readministration of 2.5 pg/cell EMD-LNPs 48 h after the initial dose also did not cause cytotoxicity (Figure S4A–D), even though emerin levels were significantly elevated after the second EMD-LNP treatment, as expected (Figure S4E).

### 2.4. EMD-LNPs Rescue Differentiation of Emerin-Null Myogenic Progenitors

**Figure 3:**
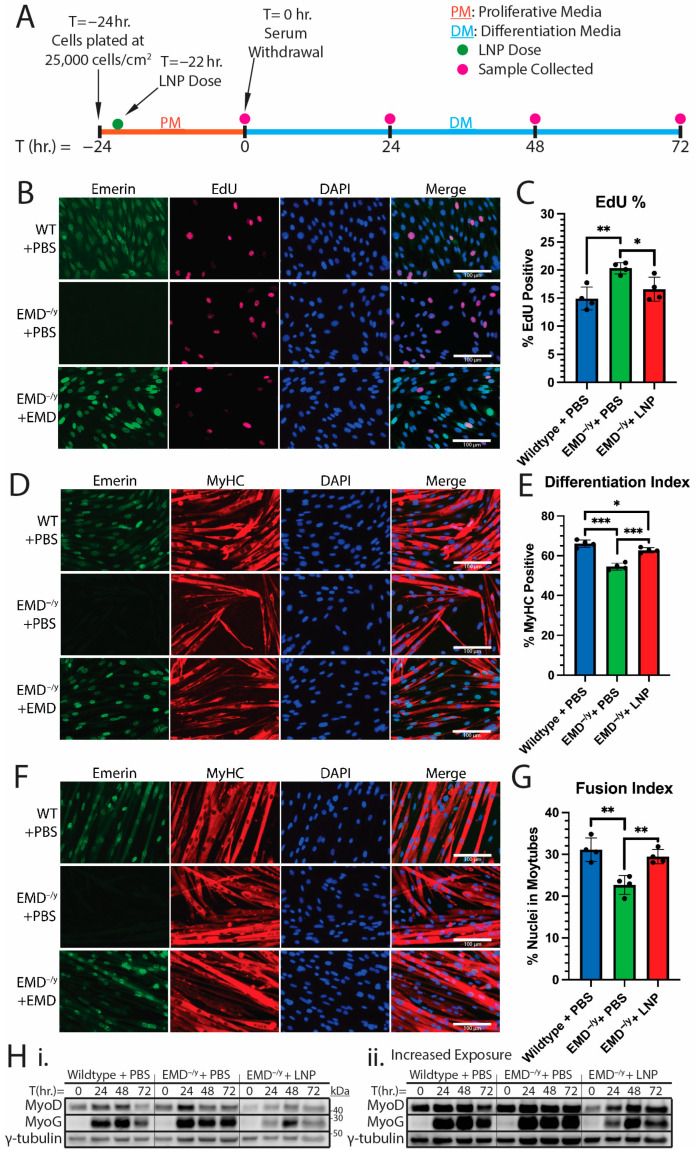
EMD-LNPs rescue differentiation of emerin-null myogenic progenitors. (**A**) Line graph showing the timing of LNP treatment (green dot), differentiation induction, and sample collection (pink dot). Wildtype myogenic progenitors treated with PBS (WT + PBS), emerin-null progenitors treated with PBS (EMD^−/y^ + PBS), or emerin-null progenitors treated with 2.5 pg/cell of EMD-LNPs (EMD^−/y^ + LNP) were incubated for 22 h and induced to differentiate. (**B**) After 24 h, the cells were incubated with EdU for 2 h. The cells were fixed and incubated with emerin antibodies. EdU, red; emerin, green; DAPI, DNA (blue). (**C**) Quantification of EdU-positive nuclei. (**D**,**F**) Immunofluorescence microscopy was performed after 48 h (**D**) or 72 h (**F**) to detect MyHC (red) or emerin (green). DAPI, DNA (blue). (**E**) The differentiation index was quantified by determining the percentage of MyHC-positive cells. (**G**) The fusion index represents the percentage of nuclei present in a shared (≥3 nuclei/cell) MyHC-positive cytoplasm. (**H**) Western blot images of myogenesis markers in differentiating myogenic progenitors. Representative images of normal exposure (i) or increased exposure (ii) are shown. Scale bars represent 100 µm. Error bars represent S.D. (*n* = 4; ≥100 nuclei per biological replicate); * *p* ≤ 0.05; ** *p* ≤ 0.01; *** *p* ≤ 0.001.

We showed that the stable transduction of wildtype emerin into emerin-null H2K myogenic progenitors rescued cell cycle withdrawal, the differentiation index, and fusion index to wildtype levels [5,10]. Therefore, we wanted to assess the ability of EMD-LNPs to rescue differentiation, a requirement for therapeutic use. We performed differentiation assays (Figure 3A) on emerin-null myogenic progenitors treated with emerin LNPs or PBS, as previously described [5,6,10]. We analyzed cell cycle withdrawal using EdU incorporation to determine the proportion of actively dividing or recently divided cells (Figure 3B). Wildtype H2K myogenic progenitors rapidly exit the cell cycle with 14.9% ± 2.0% still cycling at 24 h post-differentiation induction (Figure 3C). Alternatively, 20.3% ± 1.0% of emerin-null myogenic progenitor nuclei were positive for EdU after PBS treatment, showing impaired cell cycle exit when compared to wildtype (Figure 3B,C). The treatment of emerin-null progenitors with emerin LNPs rescued cell cycle exit, as 16.6% ± 2.1% of these nuclei were EdU-positive (Figure 3B,C).

At 48 h after differentiation induction, wildtype or emerin-null cells dosed with 2.5 pg/cell EMD-LNP or PBS were fixed and stained for myosin heavy chain (MyHC) to measure the differentiation index (Figure 3D,E). The differentiation index is defined as the number of cells with MyHC-positive cytoplasm compared to the total number of cells [45]. As expected, 54.6% ± 1.7% of emerin-null progenitors dosed with PBS expressed MyHC compared to 66.1% ± 1.8% of wildtype progenitors (Figure 3D,E), showing impaired differentiation. Significantly more emerin LNP-dosed emerin-null progenitors expressed MyHC (62.7% ± 1.3%; Figure 3D,E), demonstrating a rescue of differentiation by EMD-LNPs.

The fusion index was measured 72 h after differentiation induction to assess myoblast fusion. The fusion index is defined as the number of nuclei within myotubes (cells with ≥3 nuclei in a shared MyHC-positive cytoplasm) divided by the total number of nuclei [45]. In total, 31.1% ± 2.8% of wildtype nuclei were present in myotubes (Figure 3F,G). Emerin-null progenitors had a significantly reduced fusion index, with 22.7% ± 2.3% of nuclei present in myotubes (Figure 3F,G). Treatment with emerin LNPs increased the fusion index to 29.4% ± 1.7% (Figure 3F,G). Therefore, our data suggests that the treatment of emerin-null myogenic progenitors with 2.5 ng/cell EMD-LNPs rescues myogenic differentiation.

We also monitored the temporal expression of the myogenic markers myoblast determinant protein (MyoD) and myogenin (MyoG) upon treatment with PBS or EMD-LNPs (Figure 3H). Wildtype myogenic progenitors show an increasing expression of MyoD from 0 to 48 h. EMD^−/y^ myogenic progenitors showed an increased expression of MyoD at T = 0 hrs that peaked at 24 h. The treatment of EMD^−/y^ myogenic progenitors with EMD-LNPs rescued the temporal expression of MyoD back to wildtype (Figure 3H). Wildtype progenitors expressed MyoG at 24 h through 72 h, with peak expression at 48 h. In EMD^−/y^ progenitors, MyoG was expressed highly at 24 h, but unlike wildtype progenitors, MyoG expression levels were maintained at this level through 72 h. Treatment of EMD^−/y^ myogenic progenitors with EMD-LNPs rescued the temporal expression of MyoG back to wildtype expression.

### 2.5. EMD-LNPs Increase Repressive Histone Modification Levels

**Figure 4:**
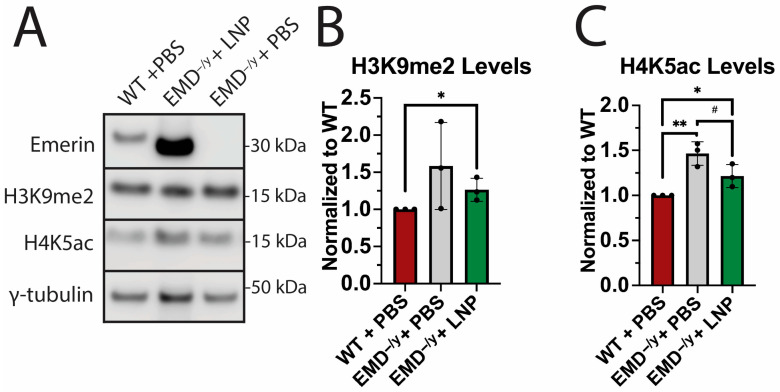
EMD-LNPs partially rescue H4K5ac and H3K9me2 levels in emerin-null myogenic progenitors. Whole-cell lysates were collected from wildtype cells treated with PBS (WT + PBS), emerin-null cells were treated with 2.5 pg/cell of EMD-LNPs (EMD^−/y^ + LNP), and emerin-null cells were treated with PBS (EMD^−/y^ + PBS) 24 h after treatment. (**A**) Western blotting for emerin, H3K9me2, H4K5ac, and *γ*-tubulin. (**B**,**C**) Quantification of H3K9me2 and H4K5ac Western blots. Levels of each protein were normalized to *γ*-tubulin and then normalized to wildtype cells. Error bars represent S.D. (*n* = 3); # *p* ≤ 0.08; * *p* ≤ 0.05; ** *p* ≤ 0.01.

Our previous data suggests that the coordinated temporal expression of differentiation genes is dysregulated in emerin-null progenitors [5,46]. Previous studies also suggest this may result from the defective genomic reorganization of these genomic loci during differentiation [47]. Thus, we tested if emerin LNPs affect genomic organization dynamics by altering histone modifications, as was seen for the stable expression of wildtype emerin in emerin-null myogenic progenitors [9]. We examined H4K5ac and H3K9me2, since they are implicated in repressive genomic organization at the nuclear envelope. We dosed proliferative emerin-null myogenic progenitors with 2.5 pg/cell for 22 h and measured the levels of H3K9me2 and H4K5ac (Figure 4A). EMD-LNP treatment increased H3K9me2 levels by 26.5% ± 15.5% compared to wildtype progenitors (Figure 4B). Compared to emerin-null PBS controls, LNP-treated emerin-null myogenic progenitors had a 20.5% ± 10.0% reduction in H4K5ac levels (Figure 4C).

## 3. Discussion

Here we showed that EMD-LNPs can successfully deliver mRNA cargo to myogenic progenitors. Upon treatment, emerin is expressed at high levels, with peak protein expression occurring at 24 h, which progressively decreases over time. Importantly, emerin expression levels remained at or above endogenous levels in wildtype cells after 4 days. We also established the dose at which EMD-LNPs become cytotoxic to be ≥15 pg/cell—much greater than the safe dose of 2.5 pg/cell used in this analysis. We have previously shown that the expression of human wildtype emerin in emerin-null myogenic progenitors rescues differentiation to wildtype levels [6,10]. Here we show that the treatment of emerin-null cells with EMD-LNPs for 24 h prior to differentiation induction rescued emerin-null myogenic progenitor differentiation. The rescue of emerin-null progenitor differentiation by EMD-LNPs was similar to the rescue seen with transfected or transduced emerin plasmids [6,10]. We should note that LNP treatment does not appear to be affected by cell attachment. We found that two hours of progenitor attachment was sufficient for delivering EMD-LNPs with maximal emerin expression. Furthermore, differentiation is not induced for 22 h after this, at which time all cells have spread and have been attached for at least 10–12 h. Thus, this treatment regimen was expected to have no effect on differentiation, and it did not, as wildtype treated with PBS alone behave similarly to wildtype untreated cells (Figure 3). This also allowed us to induce differentiation when emerin levels were highest (Figure 1). Because the skeletal muscle pathology of EDMD1 arises, at least in part, from poor myogenic differentiation, we predict that EMD-LNP treatment could provide an opportunity to design a therapeutic approach for ameliorating the skeletal muscle symptoms of EDMD1 patients.

Emerin function is important for the transcriptional reprogramming [4] that occurs during myogenic differentiation [48,49]. Chromatin-containing genes that are turned off will often become heavily deacetylated, while those being turned on will become more acetylated [50,51]. The interaction of emerin with HDAC3 activates its catalytic activity, leading to reduced H4K5ac levels [9]. Supporting this interaction as important for differentiation, emerin-null myogenic progenitors have increased levels of H4K5ac [9]. Importantly, targeting H4K5ac levels by an HDAC3 activator (theophylline) or inhibitors of H4K5-specific histone acetyltransferases rescued the differentiation and fusion indices of emerin-null myogenic progenitors [47,52,53]. Emerin-null myogenic progenitors treated with the HDAC3 activator caused the reorganization of specific stem cell and myogenic gene loci to resemble a wildtype progenitor genomic organization [47]. Here we also observed significant reductions in H4K5ac levels of emerin-null progenitors dosed with EMD-LNPs. We predict that reduced H4K5ac levels are also a direct result of the emerin–HDAC3 interaction, resulting in the rescue of the dynamic genomic reorganization during myogenic differentiation.

This data supports the use of emerin LNP as a potential therapeutic intervention for EDMD1 that requires more study to ascertain its effectiveness and feasibility. Other gene therapy interventions to treat muscular dystrophies are currently in development, which include those that utilize adeno-associated viruses (AAVs) targeted to muscle cells (myo-AAV) to deliver and integrate exogenous DNA into cells [31,54]. LNP technology may be advantageous over Myo-AAVs for a couple of reasons. First, EMD-LNPs do not rely on successful genomic integration. Secondly, myo-AAVs need to successfully infect cells by evading immunological response and often result in production of neutralizing antibodies [55]. LNP immunogenicity is lower than that of AAVs and enables repeated dosing [56]. Lastly, LNPs possess more tunable dynamics in terms of lipid composition and lipid molar ratios for targeted delivery to tissues [57].

Importantly, recent clinical results showed that LNPs were effective in restoring metabolic function in patients with propionic acidemia [58,59], demonstrating the utility of LNP platforms to restore cellular function in addition to use as a vaccine. Another LNP-based treatment, Onpattro^®^, is FDA-approved and used clinically for the treatment of hereditary ATTR amyloidosis [37]. However, LNPs are not without drawbacks entirely. One shortcoming of utilizing LNPs in the clinic would be the frequency of the dosing regimen for EDMD1 patients, since emerin expression waned over 4–7 days in this study. Importantly, redosing with emerin after 2 days resulted in efficient emerin expression with no toxicity. This will serve as a guide when developing a dosing regimen in mouse models that is efficacious and non-toxic to determine feasibility in vivo. This can then be followed by allometric scaling for human dosing, which has been accomplished for other LNP-based mRNA treatments [60]. Clinical trials will then be necessary to determine LNP efficacy and toxicity in human patients. We acknowledge that repeated dosing with LNPs may carry immunogenicity risks and will likely require thorough preclinical validation. However, it is generally understood that LNPs are immunologically inert, and the clinical precedence of repeated dosing safety has been established with FDA-approved treatments like Onpattro^®^.

While in vitro models are limited and may not translate directly in a biological context, we anticipate testing functional assays in the future. For example, contractility assays may be performed after dosing to test functional rescue, as has been performed in iPSCs derived from patients with *LMNA* mutations [61]. It has been shown that the downregulation of emerin disrupts actomyosin cytoskeleton dynamics upon cyclic stretching in fibroblasts [62]. In our case, contractility assays could provide crucial insight into functional rescue by dosing with EMD-LNPs. We anticipate that the treatment of emerin-null cells would rescue deficits in contractility magnitude, as emerin is important for actin organization [63]. Another future direction of EMD-LNPs will be testing its efficacy and toxicity in a human myogenic line, such as muscle stem cells derived from human iPSCs (hiPSCs). Recently, EDMD1 hiPSCs have been established as a new model for EDMD1 [64]. The EDMD1 hiPSC model may be more relevant for evaluating differentiation rescue, as our EMD-LNPs encapsulate human emerin mRNA. We anticipate EMD-LNPs will rescue the differentiation deficits in EDMD1 patient-derived iPSCs.

While the in vitro data presented here is promising, it will be important to test EMD-LNP utility in vivo. Mice are mildly affected by the loss of emerin, with cardiac dysfunction occurring in older mice (<40 weeks) [65]. The absence of the EDMD1 phenotype in emerin-null mice is likely due to functional redundancy with other LEM (LAP, emerin, Man1) proteins. Supporting this hypothesis, double-knockout mice lacking both emerin and LAP1 exhibit severe phenotypes consistent with EDMD [66]. LAP1-null mice exhibit less severe skeletal muscle pathology [66,67]. Thus, testing EMD-LNP dosing in emerin and LAP1 double-knockout mice would provide an in vivo model for whether EMD-LNPs rescue skeletal muscle and/or cardiac dysfunction. Based upon our in vitro results, we anticipate a restored function of emerin and LAP1 double-knockout skeletal muscle upon treatment with EMD-LNPs. These studies would include testing EMD-LNP efficacy in mice for muscle regeneration after cardiotoxin injection, monitoring satellite cell quantity, and monitoring skeletal muscle fiber architecture for a range of EMD-LNP doses. Functional rescue would also be tested by performing treadmill exercise tests, grip strength tests, and an in situ analysis of isolated muscles after EMD-LNP treatment. The dosing regimen for robust emerin expression and functional rescue would also be empirically determined. Future work will also need to determine if treatment is most feasible with intramuscular injections where LNPs are to be targeted.

The data presented within this study are a foundation for future studies utilizing EMD-LNPs for clinical applications with EDMD1 patients and potentially patients with other forms of EDMD.

## 4. Materials and Methods

### 4.1. Myogenic Progenitor Cell Lines

Myogenic progenitors from H2K wildtype and EMD^−/y^ mice were a generous gift from Tatiana Cohen and Terence Partridge (Children’s National Medical Center, Washington, DC, USA). Emerin-null H2K mice were generated by crossing emerin-null and H-2K^b^tsA58 mice [68,69]. Extensor digitorum longus muscles were isolated and incubated in DMEM supplemented with 2 mg/mL collagenase (Sigma, product #C0130; St. Louis, MO, USA) for 1–2 h at 35 °C. Individual fibers were then isolated, and each fiber was transferred serially through 2–4 Petri dishes containing DMEM to select undamaged fibers. Undamaged fibers were placed into matrigel-coated dishes containing DMEM, 0.5% chick embryo extract (Accurate Chemical #CE6507; Carle Place, NY, USA), 2% L-glutamine (Invitrogen #25030-081; Carlsbad, CA, USA), and 1% penicillin/streptomycin (Invitrogen #15140-122; Carlsbad, CA, USA) for 3–4 h at 37 °C. They were then transferred to matrigel-coated dishes with DMEM consisting of 20% heat-inactivated FBS (ThermoFisher Scientific #16000069; Waltham, MA, USA2% chick embryo extract (Accurate Chemical #CE6507; Carle Place, NY, USA), 2% L-glutamine (ThermoFisher Scientific; Waltham, MA, USA), 1% penicillin/streptomycin (ThermoFisher Scientific; Waltham, MA, USA), and 20 units/mL *γ*-interferon (MilliporeSigma; Burlington, MA, USA)) to isolate myogenic progenitors. The fibers were incubated for 24–48 h at 33 °C and 10% CO_2_. After the attachment of a single myogenic progenitor to the well, the fiber was removed and the myogenic progenitor was allowed to proliferate in proliferation media for another 48 h at 33 °C and 10% CO_2_. After 48 h, about 200 myogenic progenitors are to be expected. Myogenic progenitors were then split and maintained in proliferation media at 33 °C and 10% CO_2_. Cells between passages 4–10 were used for these studies.

### 4.2. Cell Culture

Proliferating myogenic progenitors were collected and counted by a Countess^®^ II FL Automated Cell Counter (ThermoFisher Scientific; Waltham, MA, USA). Cells were plated at 650 cells/cm^2^ and incubated in proliferative media consisting of high-glucose DMEM (ThermoFisher Scientific; Waltham, MA, USA) supplemented with 2% chick embryo extract (Accurate Chemical; #MDL-004-E; Carle Place, NY, USA), 20% heat-inactivated FBS (ThermoFisher Scientific; #16000069; Waltham, MA, USA), 1% penicillin–streptomycin (ThermoFisher Scientific; Waltham, MA, USA), 2% L-glutamine (ThermoFisher Scientific; Waltham, MA, USA), and 20 units/mL *γ*-interferon (MilliporeSigma; Burlington, MA, USA) at 33 °C and 10% CO_2_. For differentiation experiments, cells were plated at 25,000 cells/cm^2^ in proliferative media at 33 °C and 10% CO_2_ overnight. Cells were washed once with phosphate-buffered saline (PBS) and incubated with differentiation media consisting of high-glucose DMEM, 2% horse serum (ThermoFisher Scientific; #16050114; Waltham, MA, USA, and 1% penicillin/streptomycin at 37 °C and 5% CO_2_.

### 4.3. Formulation of EMD-LNPs

C12-200 was purchased from MedChem Express (Monmouth Junction, NJ, USA) and all other lipid components, including DOPE, cholesterol, and DMPE-PEG2000, were purchased from Avanti Polar Lipids Inc. (Birmingham, AL, USA). Codon-optimized luciferase and emerin mRNA (NM_000117.3) were purchased from the Engineered mRNA and Targeted Nanomedicine core facility at the University of Pennsylvania (Philadelphia, PA, USA). The mRNA was designed with 1-methylpseudouridine modifications and was co-transcriptionally capped and purified using cellulose-based chromatography. LNPs were formulated by micropipette mixing of an ethanol phase containing the lipid components and an aqueous phase containing the mRNA and citrate buffer (pH 3) at a 1:3 volumetric ratio. The formulation contained a molar ratio of 35% ionizable lipid, 10% phospholipid, 1.5% DMPE-PEG, and 53.5% cholesterol. The mRNA ionizable lipid weight ratio was 1:10. After synthesis, the LNPs were dialyzed against 1X PBS for 2 h and sterile filtered using 0.2 µm syringe filters.

### 4.4. Characterization of EMD-LNPs

Dynamic light scattering (DLS) measurements, including the hydrodynamic diameter and polydispersity index, were taken with a Malvern Panalytical Zetasizer Nano ZS (Malvern, UK). LNP samples were diluted 1:100 with PBS in cuvettes before taking DLS measurements. LNP encapsulation efficiencies were calculated with the Promega QuantiFluor^®^ RNA System kit (Madison, WI, USA). Briefly, LNPs were diluted in 1X TE buffer at a 1:100 ratio in two tubes, with one tube containing 1% Triton X-100. Samples were then heated to 37 °C under agitation (300 rpm) for 10 min. A standard curve of mRNA was prepared to quantify encapsulation within LNPs as per the manufacturer’s instructions. Once cooled to room temperature, LNP samples and standards were plated in a black 96-well plate in triplicate. Fluorescent dye was then added to each well, and the fluorescent signal was read on a plate reader (excitation: 494 nm; emission: 540 nm).

### 4.5. Differentiation Assay

Wildtype and EMD^−/y^ myogenic progenitors were counted and plated in 12-well dishes (Falcon) at a density of 25,000 cells/cm^2^ and grown in proliferative conditions for 2 h. Cells were then dosed at a concentration of 2.5 pg/cell and incubated overnight. Cells were then washed and induced to differentiate (t = 0 h) by replacing with differentiation media and grown in differentiation conditions. Plates were grown for 72 h and samples were collected every 24 h. The ClickIt EdU reaction kit (ThermoFisher Scientific #C10640; Waltham, MA, USA) was used to determine cell cycle withdrawal. After 22 h in differentiation conditions, EdU was added to cells and allowed to incubate for two hours. Samples were washed once with PBS, fixed with 3.7% formaldehyde for 15 min, permeabilized in 0.2% triton X-100 for 20 min, and stained for EdU incorporation as per the manufacturer’s instructions. To determine the myogenic differentiation index and fusion index, cells were grown for 48 or 72 h, respectively, in differentiation conditions, then fixed and permeabilized as previously described. Cells were blocked with 3% BSA in PBS at room temperature for at least 1 h with shaking. Cells were washed and incubated with a myosin heavy chain (MyHC) antibody (1:20; Santa Cruz Biotechnology; #SC-376157; Dallas, TX, USA) for 2 h at room temperature with shaking. Cells were then washed and incubated with secondary antibody anti-mouse Alexa-594 (Invitrogen #A-11032; Carlsbad, CA, USA). Nuclei were stained with 0.2 µg/mL DAPI in PBS for 5 min at room temperature with shaking. Images were taken with a 40× objective (N.A. = 0.65) using an Evos FL Auto 2 fluorescent microscope. Cell cycle withdrawal was calculated by determining the ratio of EdU-positive (Alexa-647) nuclei to the total number of nuclei (DAPI). The differentiation index was calculated by determining the ratio of MyHC-positive (Alexa-594) cells to the total number of nuclei (DAPI). The fusion index was calculated by determining the ratio of nuclei (≥3) contained in a shared MyHC-positive cytoplasm to the total number of nuclei (DAPI). Images were analyzed and counted using imageJ v1.52q software.

### 4.6. Western Blotting

Whole-cell lysates were created using NuPage^TM^ LDS sample buffer (Invitrogen #NP007; Carlsbad, CA, USA). Samples were heated at 75 °C for 15 min on a heating block prior to electrophoresis. Samples were then separated on a NuPage bis-tris gel (Invitrogen #NP0321BOX; Carlsbad, CA, USA) at constant voltage for one hour. Samples were then horizontally transferred to a nitrocellulose membrane under a constant 250 mA current for 75 min on ice. Membranes were stained with ponceau S to verify transfer efficiency. Membranes were washed with PBS containing 0.1% triton X-100 (PBST) and blocked with 5% milk (Giant Food Stores; Carlisle, PA, USA) diluted into PBST for one hour at room temperature or overnight at 4 °C. Membranes were stained with primary antibodies against emerin (1:3000; ProteinTech #10351-1-AP; Rosemont, IL, USA), *γ*-tubulin (1:10,000; Invitrogen #MA1-850; Carlsbad, CA, USA), MyoD (1:200; Santa Cruz #sc-304; Dallas, TX, USA), MyoG (1:250: Abcam #ab1835; Cambridge, UK), H3K9me2 (1:1500; Active Motif #39239; Carlsbad, CA, USA), or H4K5ac (1:1500; Sigma-Aldrich #07-327; St. Louis, MO, USA) diluted in 0.5% milk in PBST. All primary antibodies were incubated for one hour at room temperature with shaking or overnight at 4 °C with shaking. Membranes were washed at least 3 times for 5 min in PBST and incubated with HRP-conjugated secondary antibodies against mouse or rabbit for one hour at room temperature. Membranes were then washed at least 3 times in PBST and incubated for one minute in SuperSignal^TM^ West PicoPlus chemiluminescent substrate (Life Technologies #34580; Carlsbad, CA, USA) before visualizing on a Li-Cor Odyssey Fc imager. Densitometry analysis was performed in Li-Cor Image Studio v6.1 software. Bands were normalized to *γ*-tubulin as a loading control. All washing and incubation steps were performed with moderate shaking.

### 4.7. Immunofluorescence

Myogenic progenitors were grown in 48-well dishes and dosed with EMD-LNPs or PBS. Cells were fixed in 3.7% formaldehyde for 15 min. Cells were washed and permeabilized with 0.2% triton X-100 for 20 min. Cells were washed and blocked with 3% BSA in PBS for 1 h. The primary emerin antibody (1:500; ProteinTech #10351-1-AP; Rosemont, IL, USA) was diluted in blocking buffer and incubated for 2 h at room temperature or overnight at 4 °C. Cells were washed 3 times with PBS for 5 min and allowed to incubate with Alexa Fluor^TM^-conjugated secondary antibodies against rabbit (Invitrogen; Carlsbad, CA, USA) for one hour at room temperature shielded from light. All washing and incubation steps were performed with moderate shaking.

### 4.8. Cell Viability Assay

EMD^−/y^ myogenic progenitors were plated in a 96-well opaque-walled dish with clear bottoms at a concentration of 1000 cells/well. The cells were allowed to adhere to the plate for 2 h under proliferative conditions. Cells were then dosed with PBS, luciferase mRNA LNPs (LUC-LNPs), or EMD-LNPs at varying concentrations and were grown overnight in proliferative conditions. Cells were washed twice with PBS and replaced with proliferative media (no LNP) containing PrestoBlue reagent and were allowed to incubate for 1 h at 33 °C and 10% CO_2_. The plate was then analyzed for fluorescence on a Flex Station 3 plate reader and a background of PrestoBlue media-only was subtracted from each readout. Following fluorescence detection, cells were washed and replaced with proliferative media and grown 24 h in proliferative conditions. This process was repeated for 2 additional days.

### 4.9. Statistical Analyses

Statistical analyses were performed using GraphPad Prism 10.0 software. All data are represented as mean ± standard deviation (S.D.), unless otherwise noted. For cytotoxicity assays, a 2-way ANOVA with Tukey’s multiple comparisons post hoc analysis was used to determine significance. For differentiation assays and densitometry analyses, Student’s *t*-test was used to determine significance. Significance is defined as *p* ≤ 0.05. All experiments were performed with a minimum of three biological replicates.

## Data Availability

Datasets are available upon request from the authors.

## Supplementary Materials

The following supporting information can be downloaded at: https://www.mdpi.com/article/10.3390/ijms26167774/s1.
