# Supplementary material for: Development of Emerin mRNA Lipid Nanoparticles to Rescue Myogenic Differentiation
Source: Int J Mol Sci. 2025 Aug 12;26(16):7774. doi: 10.3390/ijms26167774 (PMC12386347; doi:10.3390/ijms26167774)
Supplement: Supplementary file 1 [file ijms-26-07774-s001.zip › ijms-3723263-supplementary.pdf]

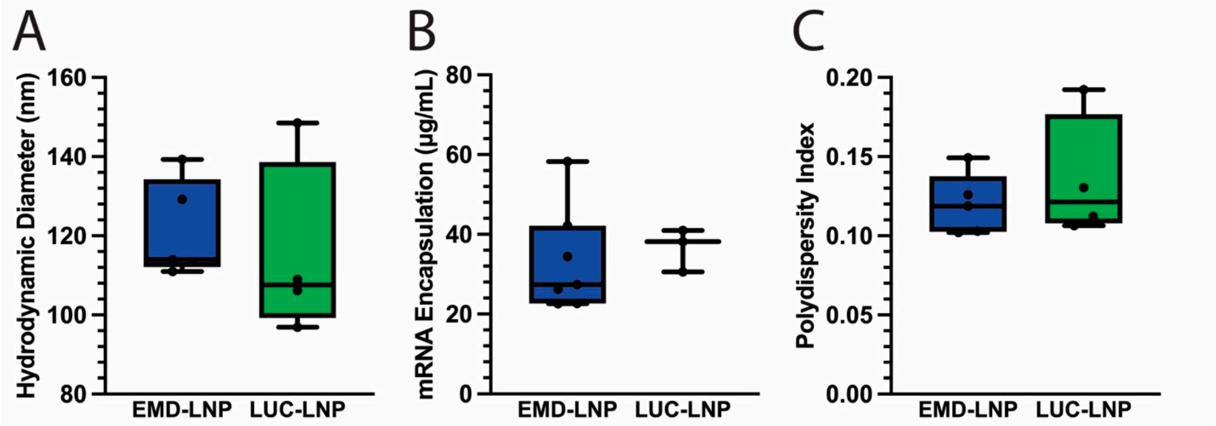

Supplemental Figure 1. LNP formulation and characterization: Characterization of emerin and luciferase mRNA LNPs including (A) hydrodynamic diameter, (B) mRNA encapsulation amount, and (C) polydispersity index. Line represents median value. Bars represent minimum and maximum values (N≥4).

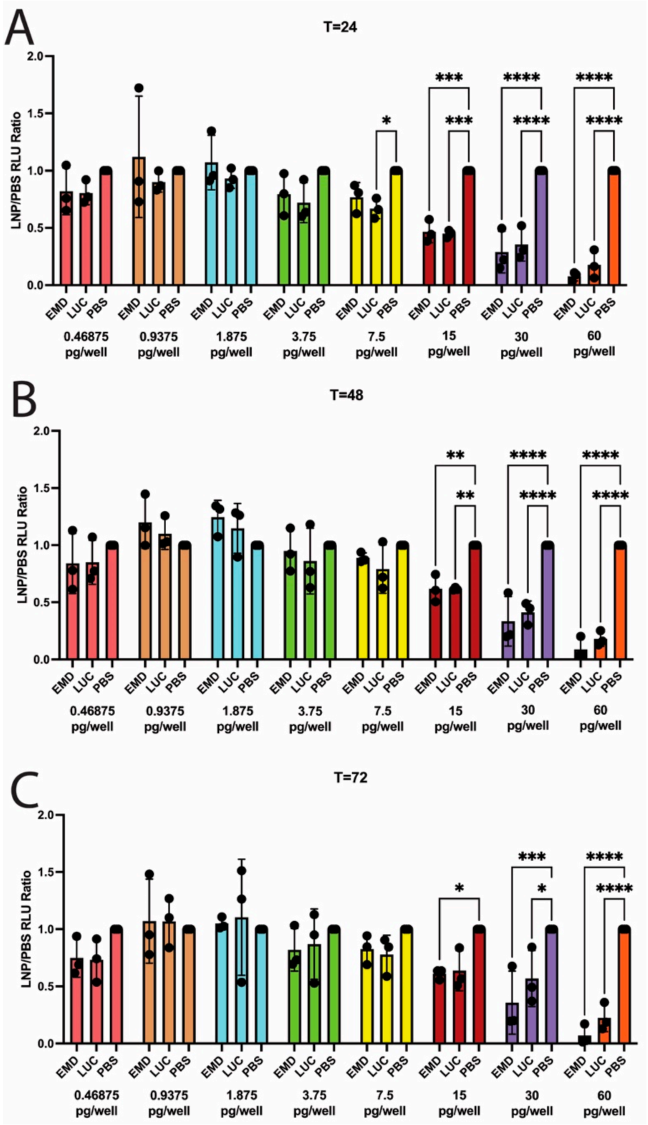

Supplemental Figure 2. Full LNP dosing regimen used to monitor cell viability of emerin-null myogenic progenitors. PrestoBlue cell viability assays were used to measure cellular metabolism in proliferating myogenic progenitors treated with increasing concentrations of PBS (PBS), EMD-LNP (EMD), or luciferase mRNA LNP (LUC). Measurements were recorded every 24 hours for 72 hours, and relative light units (RLUs) were normalized to PBS: (A) 24 hours after

LNP incubation; (B) 48 hours after LNP incubation; (C) 72 hours after LNP incubation. Error bars represent S.D. (N=3); * p≤0.05; ** p≤0.01; *** p≤0.001; **** p≤0.0001.

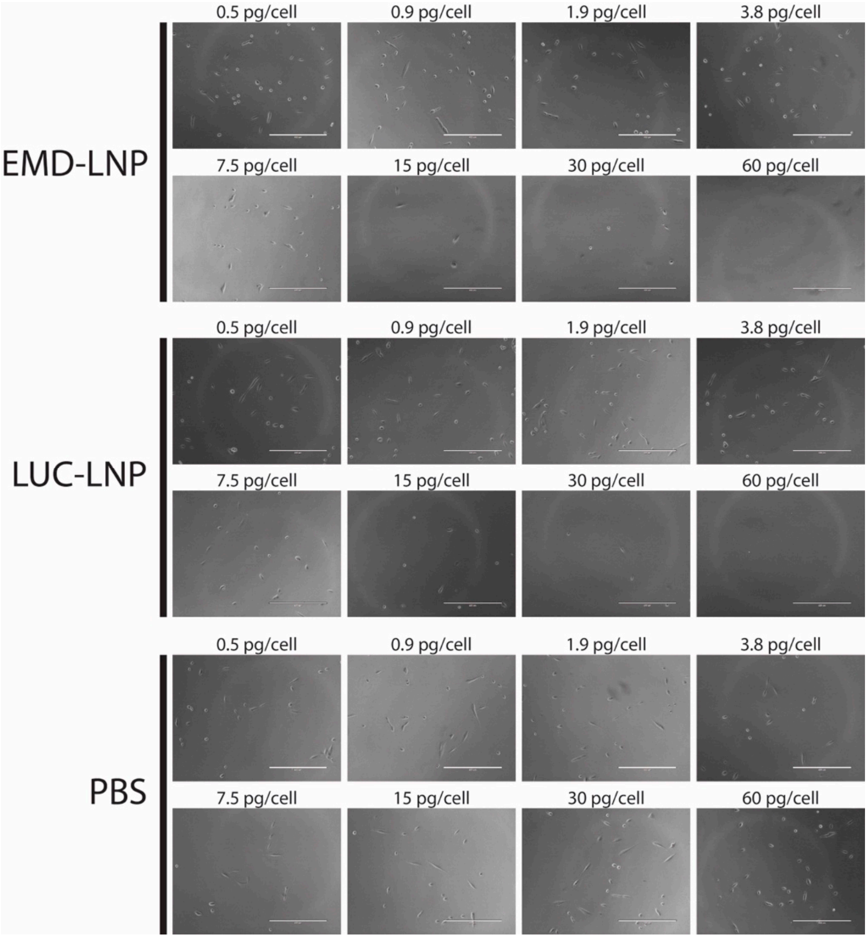

Supplemental Figure 3. Brightfield images of emerin-null myogenic progenitors treated with EMD-LNP, LUC-LNP, or PBS. Emerin-null cells were treated with varying concentrations of LNPs, and images were acquired after 24 hours. Scale bars represent 400 μm.

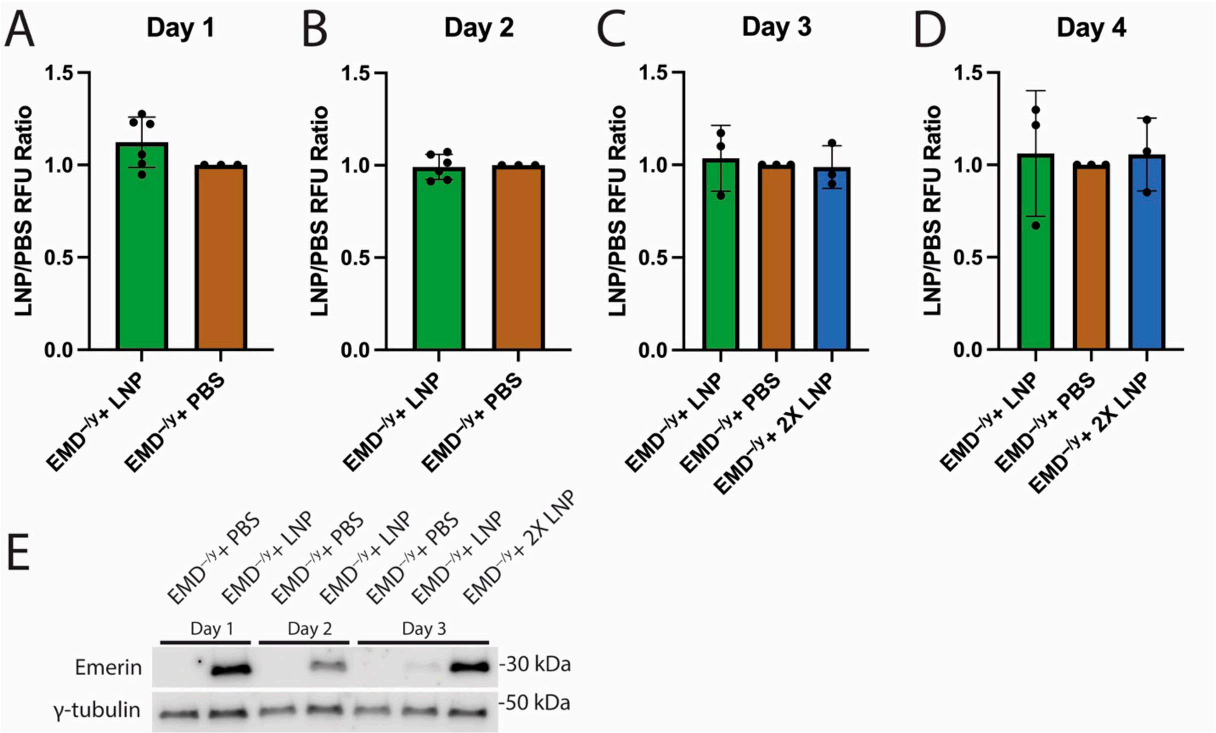

Supplemental Figure 4. Re-dosing EMD-LNPs is not cytotoxic. (A,B) Emerin-null cells were dosed with PBS (EMD$^{-/y}$ + PBS) or EMD-LNPs (EMD$^{-/y}$ + LNP; 2.5 pg/cell) at T=0 and monitored for cytotoxicity (N≥3). (C,D) Cells were re-dosed with 2.5 pg/cell EMD-LNPs (EMD$^{-/y}$ + 2X LNP) at day 2 (N=3). (E) Western blotting showing emerin levels can be restored after re-dosing with EMD-LNPs. Error bars represent S.D.
